# Editorial: Age-related hearing loss: from pathogenesis to therapy and psychiatric impact

**DOI:** 10.3389/fnagi.2026.1843137

**Published:** 2026-04-15

**Authors:** Agnieszka J. Szczepek, Yan Sun

**Affiliations:** 1 Department of Otorhinolaryngology, Head and Neck Surgery, Charité-Universitätsmedizin Berlin, Corporate Member of Freie Universität Berlin and Humboldt Universität zu Berlin, Berlin, Germany; 2 Faculty of Medicine and Health Sciences, University of Zielona Góra, Zielona Gora, Poland; 3 Department of Otolaryngology and Head and Neck Surgery, Yantai Yuhuangding Hospital, Qingdao University, Yantai, Shandong, China; 4 Shandong Provincial Key Laboratory of Neuroimmune Interaction and Regulation, Yantai, Shandong, China; 5 Shandong Provincial Clinical Research Center for Otorhinolaryngologic Diseases, Yantai, Shandong, China; 6 Yantai Key Laboratory of Otorhinolaryngologic Diseases, Yantai, Shandong, China

**Keywords:** age-related hearing loss (ARHL), central auditory processing, cognitive decline and mental health, hearing rehabilitation and prevention, presbycusis classification

Age-related hearing loss (ARHL, presbycusis) is an acquired, bilateral, usually progressive sensorineural hearing impairment. It occurs in middle to late life due to the combined effects of lifelong exposures and biological aging, leading to decreased hearing ability and communication difficulties. ARHL is one of the most common chronic conditions among older adults and a major factor in communication challenges. Its impact extends beyond audiogram results, as many older adults struggle to understand speech in noisy environments, experience increased listening effort, social withdrawal, and emotional stress. Importantly, recent studies have shown that acquired hearing impairment is considered the primary risk factor for developing dementia (Livingston et al., 2017, 2020, 2024). This Research Topic aims to integrate insights into peripheral and central auditory aging mechanisms with translational strategies for prevention and treatment, while also considering psychiatric and broader population-level impacts.

**Figure 1:**
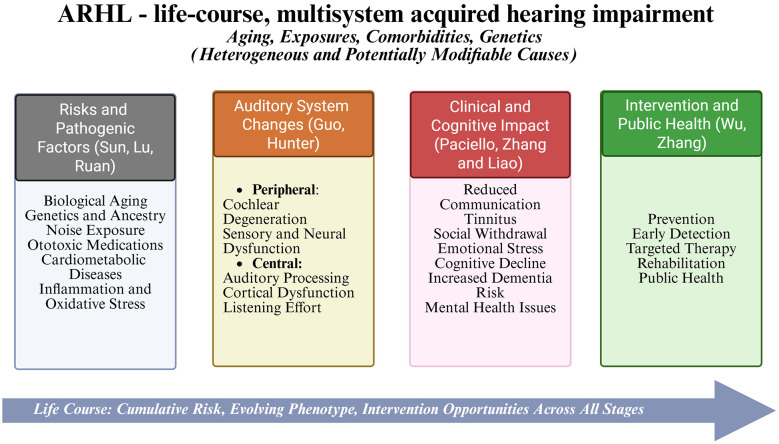
Age-related hearing loss as a life-course, multisystem condition: from redefinition to mechanisms, functional outcomes, and intervention domains (BioRender.com).

A common issue in ARHL research and treatment is the reliance on presbycusis as a quick diagnosis, which often suggests that hearing loss in older adults is unavoidable and mainly caused by aging. An international consensus from the International Consortium on Aging-Related Pathologies (ICCARP) Audiovestibular Group emphasizes that this view is not only scientifically incorrect but also detrimental to clinical practice and public health (Tsimpida et al., 2025). It may disguise the complex causes of hearing loss, such as lifelong noise exposure, ototoxic medications, and cardiometabolic diseases, leading to diagnostic complacency and weaker prevention messages. The group notes that ICD-11 currently assigns presbycusis a separate code (AB54) from “acquired hearing impairment,” creating redundancy and reinforcing age-based assumptions; they suggest removing AB54 and classifying acquired hearing loss independently of age, with additional codes to indicate severity. This perspective aligns closely with the main idea of the Research Topic: ARHL

should be viewed as a condition that occurs throughout the lifespan, with modifiable risk factors and diverse underlying mechanisms, rather than an inevitable consequence of aging. This conceptual shift and the thematic domains covered in this Research Topic are summarized in Figure 1.

In this context, where terminology and classification should promote prevention and a deeper understanding of the disease, Sun et al. analyze current pathophysiological models for ARHL and connect these mechanisms to potential treatments. Their overview concentrates on common processes such as inflammatory signaling, oxidative stress, disrupted cellular maintenance, and metabolic changes, which increase cochlear vulnerability and are linked to systemic aging. They also connect these mechanisms to intervention strategies by examining prevention and treatment options, including pharmacological, gene-based, and lifestyle modifications. Overall, the review highlights a key translational insight: successful therapy depends on accurate phenotyping of the ARHL subtype, considering individual factors and disease stage, using outcome measures that reflect real-world functions, and applying timely interventions for prevention, early treatment, and later-stage rehabilitation.

These mechanistic insights naturally raise the main question guiding current research in auditory aging: how do alterations in the peripheral system influence cognitive, emotional, and functional outcomes? Paciello et al. broaden their focus beyond the auditory periphery by investigating the cognitive effects of ARHL. They review the literature and present three interconnected models: the common-cause model (shared age-related factors), the cascade model (hearing loss leading to cognitive changes through decreased input and social effects), and the cognitive-load model (the mental resources constantly used for auditory decoding, which limits capacity for other tasks). This framework shows why mental health and cognitive function should be key endpoints in ARHL research and treatment. It also highlights that “successful therapy” should be evaluated not only based on improved hearing thresholds but also by reductions in listening effort, communication-related stress, fatigue, and social isolation, as these are equally important clinical and mechanistic outcomes.

This focus on heterogeneity involving mechanisms, trajectories, and outcomes highlights the importance of biological stratification for better prediction, targeted prevention, and treatment. Lu et al. examine the evolving genetic landscape of ARHL through bibliometric and cross-ethnic analyses. Their main point is clear: limited ancestry diversity and a lack of validation across populations impede both generalizability and fairness. As genomics shifts from discovery to risk prediction and mechanism-based interventions, having inclusive datasets and cross-population validation becomes essential, not optional. In this Research Topic, the genetic perspective supports the idea that translating pathogenesis into therapy will require comprehensive evidence that applies across diverse settings and populations.

Genetic and molecular insights must be combined with phenotypic data that demonstrate how hearing loss is experienced and processed by the central nervous system. Several studies emphasize that auditory aging often involves not only a decline in peripheral sensitivity but also limitations in central processing. Guo et al. explore temporal sound processing in the mouse auditory cortex using an envelope-steepness mapping method, revealing age-related changes in how the cortex encodes temporally structured sounds important for communication. In addition to this basic research, Hunter explores the relationship between resting-state EEG measures and age-related hearing challenges in humans. By linking hearing performance (including speech-in-noise tasks) to resting brain oscillations, this research helps develop objective neurophysiological markers that could facilitate mechanistic phenotyping and serve as endpoints in clinical trials aimed at reducing listening effort and cognitive vulnerability.

As the field moves from gaining a basic understanding to developing and applying therapies, the precision of clinical measurements becomes essential for successful translation. Wu et al. highlight a key practical concern in cochlear implant management: whether the patient's age affects the choice of inter-pulse intervals during ECAP measurements. While telemetry is often viewed as protocol-fixed, the findings indicate that using age-related parameters can reduce systematic errors and improve the interpretation of results. This study serves as a useful reminder that progress in ARHL treatment depends not only on new therapies but also on standardized, purpose-driven measurements that support personalized rehabilitation.

Importantly, ARHL and its cognitive and psychiatric effects rarely occur in isolation from other age-related health issues. Ruan et al. contextualize auditory dysfunction within multimorbidity by linking peripheral and central hearing problems to cardiometabolic multimorbidity and cognitive performance among community-dwelling older adults. Their results support a practical approach: hearing and brain health are part of a systemic risk model, indicating that prevention and risk management should target vascular and metabolic factors, as well as sensory rehabilitation.

This integrated framework emphasizes the urgency and scale of the issue at the population level. Zhang et al. quantify the increasing global burden of hearing loss among adults aged ≥60 years using Global Burden of Disease data and project further growth, highlighting the need for prevention strategies and accessible care options. Separately, Zhang and Liao explore the factors behind ARHL and tinnitus, demonstrating that their co-occurrence is associated with worse health outcomes than either condition alone. Together, these public health insights underscore that screening and management should be combined—integrating hearing care with tinnitus support and mental health attention—rather than being provided through separate pathways.

In summary, the papers in this Research Topic agree on an important point: ARHL should be recognized and treated as a complex, multisystem condition that develops over a lifetime. Progress will be quicker if we improve terminology and classification to avoid relying on age alone; combine genetics and mechanistic biology with meaningful functional phenotypes; expand biomarkers and neurophysiological measures relevant to real-world communication challenges; and integrate hearing care into prevention strategies that consider multimorbidity and mental health. We thank the authors and reviewers for advancing this comprehensive approach and hope that this Research Topic inspires translational research that connects cochlear biology to brain and psychiatric outcomes.
